# Oral Bacterial and Fungal Microbiome Impacts Colorectal Carcinogenesis

**DOI:** 10.3389/fmicb.2018.00774

**Published:** 2018-04-20

**Authors:** Klara Klimesova, Zuzana Jiraskova Zakostelska, Helena Tlaskalova-Hogenova

**Affiliations:** Laboratory of Cellular and Molecular Immunology, Institute of Microbiology of the CAS, Prague, Czechia

**Keywords:** microbiome, mycobiome, pathobiont, dysbiosis, biofilm, *Fusobacterium*, oral diseases

## Abstract

Host’s physiology is significantly influenced by microbiota colonizing the epithelial surfaces. Complex microbial communities contribute to proper mucosal barrier function, immune response, and prevention of pathogen invasion and have many other crucial functions. The oral cavity and large intestine are distant parts of the digestive tract, both heavily colonized by commensal microbiota. Nevertheless, they feature different proportions of major bacterial and fungal phyla, mostly due to distinct epithelial layers organization and different oxygen levels. A few obligate anaerobic strains inhabiting the oral cavity are involved in the pathogenesis of oral diseases. Interestingly, these microbiota components are also enriched in gut inflammatory and tumor tissue. An altered microbiota composition – dysbiosis – and formation of polymicrobial biofilms seem to play important roles in the development of oral diseases and colorectal cancer. In this review, we describe the differences in composition of commensal microbiota in the oral cavity and large intestine and the mechanisms by which microbiota affect the inflammatory and carcinogenic response of the host.

## Introduction

Both the upper and lower parts of the human digestive tract harbor a complex ecosystem of bacteria, fungi, protozoa, and viruses, referred to as the microbiome. It begins to form even before birth, in the uterus, developing for another 2–3 years after birth to become a stable, fully functioning microbiome, until the physiological changes associated with senescence lead again to substantial shifts in its composition (Adlerberth and Wold, 2009; Aagaard et al., 2014; Maffei et al., 2017). The lower part of the digestive tract gets “inoculated” every day by about 10^11^ bacteria from the oral cavity and microbial species detected in the oral and fecal microbiota overlap in about 45% of tested individuals (Socransky and Haffajee, 2002; Segata et al., 2012). Moreover, via the blood stream, these oral bacteria can disseminate all over the body. Fungal microbiota can colonize the gut perorally as well, with some strains detected in the gut likely to be contaminants from the environment or food, rather than commensals (Trojanowska et al., 2010). The composition and function of the microbiome change along the digestive tract, from the oral cavity to the rectum. These differences have been previously described in detail (Arumugam et al., 2011; Human Microbiome Project Consortium, 2012) and will be briefly outlined below.

Collectively, the genes encoded by the microbial genomes outnumber the genes in the human genome about 100-fold and this variation enables the commensal microbiota to use substrates indigestible by humans (Qin et al., 2010). Products of microbial metabolic activity include vitamins, short-chain fatty acids (SCFAs), and other compounds important for host cell metabolism and survival. Moreover, the host’s physical interaction with or sensing of the microbial components is important for proper mucosal barrier function and mucosal immune system development and homeostasis. On the other hand, recent studies have shown that some commensal microbes can under certain conditions become pathogenic – so called pathobionts. Mechanisms include expression of virulence factors, such as adhesion molecules or proteases, or formation of a biofilm, and such activity can lead to disease initiation or progression. One such example is *Escherichia coli*, a large and diverse group of various bacterial serotypes. *E. coli* strains differ in their activities and biological roles: some of them are gut commensals and commercially available probiotics, others can be pathogens causing gastrointestinal and urinary infections or pathobionts associated with inflammatory bowel disease (IBD) and colorectal cancer (CRC).

**FIGURE 1:**
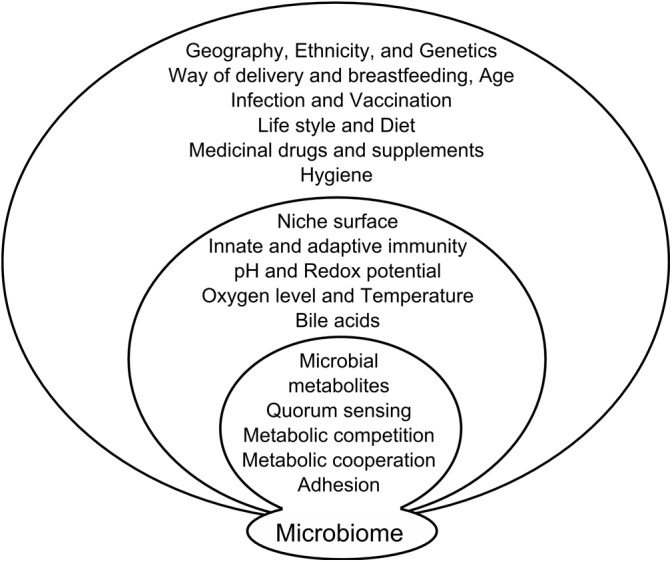
Factors influencing microbiome composition. Microbiome composition is influenced on several levels by factors from external environment, factors resulting from the interaction of microbiota with the host and mutual interaction of the microbiome components.

The jury is still out on what triggers this transformation of commensals into pathobionts; it might be that a change in the gut microenvironment simply allows the microbes to interact with the host in an aberrant way. The gut microbiota composition and function can be influenced by various factors (**Figure 1**). For instance, many substances produced by the host and secreted into gut lumen, such as antibacterial peptides, secretory IgA, mucins, cytokines, or neuromediators, can shape the microbial community. Their production depends on host gene polymorphisms and their expression in host cells is often driven by microbial stimulation, creating a positive feedback loop. Mechanisms responsible for cancer development include some of these factors but others are still under scientific investigation.

Colorectal cancer is the most prevalent type of cancer in developed and developing countries (Ferlay et al., 2015). Only a small percentage of colorectal cancers is hereditary or associated with certain predisposing conditions, such as chronic intestinal inflammation in IBD patients. The majority of cases thus represents sporadic cancers (85–95%) and can be, to some extent, influenced by environmental factors. The composition and metabolic activity of gut microbiota may be therefore a crucial component of CRC pathogenesis (Arthur and Jobin, 2011).

Findings of significant colonization of cancer tissue by microbes usually found in the oral cavity have sparked a debate about a possible involvement of oral microbiota in CRC development process. Many experimental studies have provided evidence for a significant role of microbiota in carcinogenesis. However, due to the complexity of microbial cooperation and interaction with the host, all the underlying mechanisms are yet to be elucidated. Here, we review the properties of bacterial and fungal populations inhabiting the oral cavity and gut, with emphasis on their association with CRC pathogenesis.

## Oral Microbiota

The microorganisms found in the human oral cavity are referred to as the human oral microbiome and play a crucial role in maintenance of homeostasis in the mouth. Each individual’s oral microbiome consists of a distinct set of microorganisms. The mouth supports one of the most diverse microbial communities compared with other body sites, such as the skin and vagina, due to its heterogeneity and the interrelationships between the different anatomic structures (Wade, 2013). The constantly humid environment of the oral cavity is maintained at a relatively stable temperature (34–36°C), while the varying pH levels and different types of diet contribute to the substantial microbiome variability (Marcotte and Lavoie, 1998). Habitats of the oral cavity are represented by both hard (teeth) and soft tissues (cheek, tongue, lip, gingival sulcus, attached gingiva, hard and soft palate) and their interface (subgingival and supragingival margins, and gingival crevices around teeth). The contiguous extensions of the oral cavity, including the tonsils, pharynx, esophagus, Eustachian tube, middle ear, trachea, lungs, nasal passages, and sinuses, are also colonized by the oral microbiome but the majority of studies describing the oral microbiome composition include only samples from the oral cavity (Mager et al., 2003; Aas et al., 2005). Moreover, all these structures are constantly moistened by two physiological fluids, saliva and the gingival crevicular fluid, which help to maintain the oral microbiome homeostasis by providing water, nutrients, antibodies, and antimicrobial and adherence factors (Marcotte and Lavoie, 1998).

The National Institute of Health’s Human Microbiome Project identified the most dominant phyla that account for over 95% of the entire oral microbiome: *Firmicutes*, with the *Streptococcus* as a dominant genus, *Bacteroidetes*, strongly represented by *Prevotella* and *Proteobacteria*, with highly abundant *Haemophilus*, *Fusobacteria,* and *Actinobacteria* (Human Microbiome Project Consortium, 2012; Huse et al., 2012; Zhou et al., 2013). The oral cavity displays greater alpha-diversity (species richness) than either the skin or vagina, characterized by uniform abundance of the major species (Huse et al., 2012). On the other hand, beta-diversity, i.e., differences in microbiome composition in oral sites among various subjects, is the lowest compared to the other body sites (Huse et al., 2012). Within the oral cavity, the highest microbiota richness has been found in the gingival plaque and in saliva, whereas the lowest richness has been described in keratinized gingiva (Huse et al., 2012; Zhou et al., 2013). It seems that saliva contributes unevenly to the microbial composition of different sites in the oral cavity. Due to its rapid turnover and low levels of nutrients, saliva itself does not contain a stable indigenous biota and owes its high alpha-diversity primarily to bacteria shed from other oral tissues (Mager et al., 2003). In general, species like *Streptococcus, Gemmella, Granulicatella, Veillonella,* and *Fusobacterium* can be detected across almost all oral sites, while others are represented only at one or two oral sites, e.g., *Prevotella, Bacteroides, Corynebacterium, Pasteurella,* and *Neisseria* (Huse et al., 2012). Bacterial species in the oral cavity find the most stable environment at the supragingival or subgingival tooth surfaces. These non-shedding surfaces are covered by persisting biofilms composed mainly by *Streptococcus* and *Actinomyces*, representing the earliest colonizers of teeth, and *Veillonella* (Socransky and Haffajee, 2005; Teles et al., 2013). Taken together, although most microbial species found in the oral cavity differ in their abundance, their representation and activity is more important.

## Oral Microbiome and Oral Diseases

A considerable number of oral conditions, including caries and periodontal diseases, endodontic infections, alveolar osteitis, and tonsillitis is connected to detrimental alteration in microbiota composition – a dysbiosis (Costalonga and Herzberg, 2014; Proctor and Relman, 2017). For instance, periodontal inflammation is directly induced by microbes colonizing the biofilm in the gingival sulcus and, at later stages, in the periodontal pockets. These oral biofilms contain 100s of different bacterial species in one periodontal lesion, with composition different from that in healthy periodontium (Socransky and Haffajee, 2005; Teles et al., 2013). Initially, the plaque forms only supragingivally but if it is not removed properly, after a few days it spreads below the gingival margin and into the sulcus. There, after the depletion of oxygen, a new environment is established, where anaerobic bacteria can flourish. Three most destructive anaerobic bacteria involved in severe periodontal disease, the so called “red complex,” include *Treponema denticola*, *Tannerella forsythia* and *Porphyromonas gingivalis* (Socransky et al., 1998). Aggressive form of periodontitis is further associated with *Aggregatibacter actinomycetemcomitans* (Slots, 1976). The immunopathological mechanisms in periodontal disease development and course have been recently thoroughly reviewed by Hajishengallis and Korostoff (2017). Recurrent aphthous stomatitis is also connected to changes in microbiota composition; decreased abundance of *Streptococcus salivarius* and increased *Acinetobacter johnsonii* have been linked to the disease incidence (Bankvall et al., 2014; Kim et al., 2016).

As mentioned previously, the human microbiome diversity is not limited only to bacteria but also includes fungal species. However, this oral mycobiome have been only recently characterized and despite its potential great scientific importance, we found only few studies where its composition was analyzed using high throughput sequencing. Ghannoum et al. (2010) reported that healthy oral mycobiota contained 74 culturable and 11 non-culturable fungal genera. They have revealed great inter-individual variation and proposed that the presence of certain fungal isolates (e.g., *Candida, Aspergillus, Cryptococcus*) probably predisposes the host to opportunistic infections. *Malassezia* species, previously described as commensals and pathogens of the skin and lungs, have been recently found as predominant commensals in saliva (Saunders et al., 2012; Dupuy et al., 2014). Many other species are likely still waiting to be discovered and were not detected earlier because of their special growth requirements (Nagano et al., 2010). The first evidence of interactions among members of the oral mycobiome community and their association with specific disease came from a study characterizing the oral mycobiome in HIV patients (Mukherjee et al., 2014). The authors found that a decrease in abundance of an indigenous fungus *Pichia* in uninfected individuals went hand in hand with an increase in abundance of *Candida*, suggesting an antagonistic relationship. *Pichia* inhibits *Candida* by different mechanisms, including competition for nutrients and secretion of factors that disrupt the latter’s ability to adhere, germinate, and form biofilms. Moreover, they found a negative correlation between *Candida* and *Campylobacter* in HIV-infected subjects, whereas in healthy subjects, no correlation between *Candida* and bacterial species was detected (Mukherjee et al., 2014). Recently, Peters et al. (2017) published a pilot study describing oral mycobiome in healthy subjects and those with periodontal disease. In diseased subjects, they found a slightly increased abundance of *Candida* genera which is in agreement with previous culture-based studies (Urzúa et al., 2008; Canabarro et al., 2013). Despite those first studies aiming for a deeper understanding of the factors affecting the oral mycobiota composition, information about their direct and indirect effects on human health and the interactions between the fungi and bacteria is still lacking.

The involvement of microbiota in the pathogenesis of oral diseases has been documented using gnotobiotic rat and mouse models of human diseases. Germ-free animals can be obtained by delivering the young by sterile Cesarean section and raising them aseptically in isolators for germ-free rearing. Such animals made it possible to study the effects of commensal bacteria in the oral cavity, including the effects on periodontal tissues. In germ-free mice and rats, it was demonstrated that periodontal disease and caries, similarly to other human inflammatory diseases, cannot be experimentally induced in the absence of microbiota (Heijl et al., 1980; Tlaskalova-Hogenova et al., 2004). Experimental animal models (e.g., the gavage model of periodontal disease) and *in vitro* studies revealed that certain components of oral microbiota, mainly *P. gingivalis*, play a crucial role in the innate host defense of periodontium and that dysregulation of the immune response in the presence of oral microbiota leads to inflammation and alveolar bone loss (Ivanyi et al., 1991; Darveau et al., 2012; Papadopoulos et al., 2013). The mechanisms by which microbiota triggers the pathological changes are not yet fully understood, however, new approaches promise to shed light on the role of oral microbiota (Kinane et al., 2017; Pitts et al., 2017).

## Oral Microbiome and Extra-Oral Diseases

Dysbiosis of the oral microbiome is not only connected with the incidence and maintenance of oral diseases, but has been also implicated in the pathogenesis of autoimmune, inflammatory, and neoplastic diseases (e.g., heart disease, respiratory illnesses, psoriasis, psoriatic arthritis, and carcinogenesis at various sites) (He et al., 2015; Roberts and Darveau, 2015; Egeberg et al., 2017; Ungprasert et al., 2017). Moreover, oral microbiota seems to affect the outcome of pathological pregnancy (preterm birth, abortions, etc.); reviewed by Cobb et al. (2017). Periodontal bacterial DNA has been found in atherosclerotic plaques of patients suffering from ischemic heart disease and atherosclerosis (Ford et al., 2005, 2006). Bacteria may initiate or exacerbate atherosclerotic processes through activation of innate immunity, direct involvement of mediators activated by dental plaque antigens in atheroma processes, or involvement of cytokines and heat shock proteins from dental plaque bacteria. There might be also genetic predisposing factors influencing both diseases (Bartova et al., 2014). Furthermore, patients with rheumatoid arthritis have a higher prevalence of periodontal disease and vice versa (Kasser et al., 1997; Greenwald and Kirkwood, 1999). Bacterial DNA has been detected in the synovial fluid of patients with rheumatoid arthritis or with failed prosthetic joints, suggesting the possibility of infection translocating from the periodontal tissue to the synovium (Temoin et al., 2012). The oral microbiome is not confined to spreading to contiguous epithelial surfaces, but can also be carried by the bloodstream to distant body sites, such as the heart, skin, and joints. Oral microbiota enters the bloodstream during routine daily activities like tooth brushing or through inflamed tissue in the course of oral diseases (Tomas et al., 2012). The mechanisms of dissemination of potentially pathogenic microbes from the oral cavity through bloodstream are still not clear. Potential connection with systemic low-grade inflammation has been also discussed (Potgieter et al., 2015).

In most conditions discussed so far, no particular microbe or microbes have been described as a causative agent. However, thanks to advances in molecular methods, presence of *Fusobacteria* and other oral bacteria has been recently demonstrated in various systemic pathological conditions, including digestive diseases, such as appendicitis, IBD and CRC. Moreover, a correlation of the presence of oral bacteria *P. gingivalis* and *A. actinomycetemcomitans* with an increased risk of developing pancreatic cancer has been observed in a large group of subjects with incident primary pancreatic adenocarcinoma (Fan et al., 2016). Several recent studies have repeatedly confirmed the presence of oral bacteria in the gut, especially in association with CRC mucosa. For instance, Nakatsu et al. (2015) have shown that a substantial part of gut microbiome associated with CRC is composed of oral bacteria and *Fusobacterium* in particular. Momen-Heravi et al. (2017) also proposed a role of oral bacteria in CRC development. They conducted a retrospective study in a huge cohort of women and discovered an association of periodontal disease and tooth loss with CRC morbidity and found that women with less than 17 teeth may be at a greater risk of incident CRC (Momen-Heravi et al., 2017).

## Oral Microbiota and Colorectal Carcinogenesis

It is generally accepted that the gut microbiome plays a role in CRC development. Experimental proof of gut microbiota involvement in CRC development came from gnotobiotic animal models (Reddy et al., 1975; Vannucci et al., 2008; Arthur and Jobin, 2011; Klimesova et al., 2013; Tlaskalova-Hogenova et al., 2014). Recent studies using next generation sequencing and polymerase chain reaction have shown that *Fusobacterium nucleatum* is frequently detected in stool and biopsy samples from CRC patients (Castellarin et al., 2012; Kostic et al., 2012; Flanagan et al., 2014). These adhesive, anaerobic Gram-negative bacteria attract considerable attention in search for possible mechanisms behind their inflammatory and tumorigenic activity. Some of the features of *F. nucleatum* and the host responses are already known: *F. nucleatum* modulates E-cadherin/β-catenin signaling via its FadA adhesin/invasin – a key virulence factor, and alters macrophage infiltration and methylation of the CDKN2A promoter in CRC lesions (Rubinstein et al., 2013; Park et al., 2017). It seems that Fap2 Gal-GalNAc lectin of *F. nucleatum* could be responsible for its tendency to bind to tumor cells displaying Gal-GalNAc moieties (Abed et al., 2016). Moreover, several other virulence proteins that might participate in inflammatory and neoplastic processes have been described in *F. nucleatum,* using proteomic approaches (Zanzoni et al., 2017). In the host, *F. nucleatum* activates numerous immune responses including human β-defensin production, lymphocyte apoptosis, and production of proinflammatory cytokines interleukin (IL)-6, IL-8, and TNF-α (Han, 2015).

The fact that *Fusobacterium* is highly abundant in patients with CRC led to various efforts to apply this finding for clinical purposes. For instance, a highly sensitive DNA test for *F. nucleatum* has been developed for screening and prognosis of CRC in Japan (Yamaoka et al., 2017). *Fusobacterium* has been recently shown to predict the aggressiveness and recurrence of CRC and its resistance to chemotherapy. It seems to be modifying the innate immune signaling and regulating specific microRNAs that activate the autophagy pathway (Yu et al., 2017). In connection with this finding, an argument has been made to use anti-*F. nucleatum* therapy together with chemotherapy.

The dietary patterns leading to CRC development have been studied for several decades. Recently, it was shown that individuals consuming a western-type diet have a higher incidence of *Fusobacterium*-associated CRC and that diets rich in whole grains and dietary fiber are associated with a lower risk of *F. nucleatum*-positive CRC (Mehta et al., 2017).

Despite the clinical observation of increased abundance of *Fusobacterium* in patients with CRC (especially in chemoresistant forms of CRC), direct clinical evidence of a causal relationship is still lacking. A recent study revealed that fusobacterial abundance is not significantly increased in fecal samples of patients with adenomas, implying that the relationship between *Fusobacterium* and CRC might not be causal after all. Amitay et al. (2017) hypothesize that *Fusobacterium* is more likely just a passenger colonizing the favorable niche in a gut with CRC, rather than the driver of cancer development. Experimental evidence of its potential causal role is based on *in vitro* and animal models of CRC. For instance, it has been shown that *F. nucleatum* potentiates tumorigenesis in monoassociated Apc^min/+^ mice (Kostic et al., 2013).

While *Fusobacterium* is the most studied periodontal microbe in connection to CRC, other components of the oral microbiome may also be implicated in CRC pathogenesis and will be discussed later (**Table 1**).

**Table 1:** Oral microbiota and its possible mechanisms related to tumorigenesis.

Microbial genera	Activity	Reference
*Streptococcus*	Adhesion, co-aggregation Biofilm formation Protease activity Hemolytic activity	da Silva et al., 2014 Diaz et al., 2012 Kilian et al., 1988 Wong et al., 2016
*Peptostreptococcus*	Biofilm formation Antiapoptotic effect Hydrogen sulfide production	Tsoi et al., 2017 Persson et al., 1990
*Parvimonas*	Biofilm formation Hemolytic activity Proinflammatory stimulation Hydrogen sulfide production	Wong et al., 2016 Marchesan et al., 2016 Carlsson et al., 1993
*Dialister*	Biofilm formation Proinflammatory stimulation	da Silva et al., 2014 Sousa et al., 2014
*Mogibacterium*	Biofilm formation	Casarin et al., 2012
*Fusobacterium*	Adhesion Proinflammatory stimulation Immune evasion Hemolytic activity Hydrogen sulfide production	Kostic et al., 2013 Rubinstein et al., 2013 Wong et al., 2016 Claesson et al., 1990
*Porphyromonas*	Biofilm formation Immune evasion Antiapoptotic effect Protease activity Hemolytic activity Hydrogen sulfide production Proinflammatory stimulation	Inaba et al., 2013 Mao et al., 2007 Potempa et al., 2003 Wong et al., 2016 Persson et al., 1990 Sousa et al., 2014
*Campylobacter*	Hemolytic activity Proinflammatory stimulation	Wong et al., 2016 Marchesan et al., 2016
*Candida*	Adhesion Biofilm formation Protease activity Hemolytic activity	Diaz et al., 2012 Gomes et al., 2017

## Gut Microbiome, Metabolic Activity, and Colon Carcinogenesis

Thanks to advances in high-throughput sequencing and metabolomic approaches, we have a growing understanding of the composition and metabolic activity of the microbiome associated with colorectal carcinogenesis. Studies have shown that gut microbiome differs between healthy individuals and adenoma/carcinoma patients and that microbial diversity in cancer is reduced (Peters et al., 2016). The adenoma-carcinoma sequence in CRC development suggests an associated continuous alteration of the resident microbiome, which is supported by recent research. Analyses of fecal microbiome composition in patients with adenoma have shown increased normalized abundance of genera such as *Actinomyces*, *Corynebacterium*, *Porphyromonas*, *Mogibacterium,* and *Haemophilus* when compared with healthy individuals (Peters et al., 2016; Hale et al., 2017). Cancer patients have shown further differences in fecal microbiota composition, with a marked enrichment of *Ruminococcus*, *Oscillibacter*, and *Roseburia*, and *Porphyromonas*, *Fusobacterium*, and *Peptostreptococcus*, i.e., strains associated with periodontal disease (**Table 1**) (Shen et al., 2010; Flemer et al., 2017; Liang et al., 2017).

Moreover, fecal samples reflect the microbial colonization of tissues, as biopsies from adenomas and carcinomas have been similarly different from healthy mucosa (Flemer et al., 2017). The findings that there is very little difference in microbiota composition between diseased and adjacent unaffected tissue suggest that the microbiome undergoes a systemic change, affecting the whole community (Lu et al., 2016; Flemer et al., 2017). Such results support the driver/passenger model, the idea that certain strains disturb the microbial community and the mucosal microenvironment (drivers) and such changes lead to subsequent colonization by pathobionts and pathogens (passengers) (Tjalsma et al., 2012). Both the drivers and the passengers modulate the local microenvironment through different means, such as virulence factors or metabolic activity. Drivers thus promote cancer initiation at the very beginning by their involvement in DNA damage, cell cycle regulation, apoptosis and epithelium proliferation, whereas passengers more likely promote tumorigenesis via chronic proinflammatory stimulation and direct tissue damage.

Drivers often include microbes that produce genotoxic substances, which damage DNA, or cyclomodulins, which can modulate the epithelial cell cycle – recently thoroughly reviewed by Gagniere et al. (2016) and El-Aouar et al. (2017). *Bacteroides fragilis* and *Enterococcus faecalis* have the potential to damage epithelial cells and initiate cancer formation by producing the enterotoxin fragilysin and reactive oxygen species, such as superoxide, respectively (Huycke et al., 2002; Toprak et al., 2006). Several studies confirmed that *E. coli* strains encoding the genotoxic polyketide synthase (pks) island are associated with inflamed gut mucosa and CRC (Swidsinski et al., 1998; Nougayrede et al., 2006; Arthur et al., 2012; Raisch et al., 2014). Interestingly, members of the family Enterobacteriaceae, including *E. coli*, can produce several types of genotoxins or cyclomodulins. Cytotoxic necrotizing factors and cycle-inhibiting factor modulate the cell cycle and can lead to uncontrolled proliferation or cell cycle arrest, respectively (Taieb et al., 2006; Miraglia et al., 2007). Cytolethal distending toxins, similarly to pks, induce DNA double-strand breaks and apoptosis, but can also promote proinflammatory cytokine production in the host (Blazkova et al., 2010). In summary, the interplay of these factors with gut epithelium and immune cells can promote low-grade inflammation and cancer initiation. Moreover, we can assume that these molecules represent just the tip of the iceberg of as yet unknown microbial products of gut commensals with the potential to harm the gut epithelium.

Mycobiome, i.e., the fungal microbiome, forms an integral part of the gut microbial community, although it is much less investigated then the bacterial part. The most common genera residing in a healthy gut are *Candida*, *Saccharomyces,* and *Cladosporium* (Hoffmann et al., 2013). However, some non-commensal transient fungi, acquired with food or from the environment, can be also found in fecal samples and may comprise potentially pathogenic species. Trojanowska et al. (2010) have shown that the gut is colonized also by the oral mycobiome, as they found a genetically identical *Candida albicans* strain in the mouth and colon of patients with IBD. Unfortunately, data about fungal colonization of the digestive tract in relation to neoplastic diseases are still sparse. A disruption of the bacterial and fungal community – dysbiosis, has been observed in individuals with IBD (Sokol et al., 2017), who are known to be at increased risk of CRC development. Interestingly, reduced richness and diversity has been detected not only in bacterial, but also in fungal microbiome (Chehoud et al., 2015; Liguori et al., 2016; Sokol et al., 2017). For instance, the Cystofilobasidiaceae family, *Dioszegia* genus and *Candida glabrata* have been found to be enriched in Crohn’s disease compared with healthy mucosa (Liguori et al., 2016). The only published study on fungal microbiota in CRC deals with comparison of adenomas and adjacent tissues. Luan et al. (2015) have observed an increased abundance of *Phoma* and *Candida* genera and *Candida tropicalis* in adenomas. As pathobionts, these genera may be involved in cancer initiation, but further studies are needed to investigate whether they work as drivers or passengers.

Microbiome in the colon makes use of various catabolic and anabolic pathways, which enable it to utilize a broad spectrum of substrates that are not absorbed in the small intestine. These pathways interact with the metabolism of xenobiotics and influence micronutrient bioavailability, lead to the production of essential vitamins and degradation of fibers, and regulate the secretion of various molecules (Arthur and Jobin, 2011). Different dietary components can shift the microbiome composition. For instance, a diet high in resistant starch increases the abundance of bacteria metabolizing non-digestible polysaccharides (Walker et al., 2011). Indeed, increased abundance of *Prevotella* and *Bacteroides* has been observed in individuals preferring high sugar and high protein diet, respectively (Wu et al., 2011). Our digestion pathways lack the enzymes for the degradation of resistant starch and dietary fiber but the distal gut microbiome encodes about 81 different families of glycoside hydrolases (bacterial polysaccharidases, glycosidases), which are not present in the human genome (Gill et al., 2006). The microbiome thus significantly contributes to the utilization of starch, primary fiber, host-derived secretions (mucus glycans), sucrose, and monosaccharides.

Subsequent fermentation of depolymerized molecules leads to the production of SCFAs, mainly acetate, propionate, and butyrate. Compared with other microbiomes in gene libraries, the human gut microbiome is enriched with genes involved in the pathways generating SCFAs (Gill et al., 2006). Main producers of butyrate within the human gut microbiome are *Faecalibacterium prausnitzii* and *Eubacterium rectale*/*Roseburia* group (Louis et al., 2010). SCFAs provide one of the most important sources of energy, not only for intestinal epithelial cells but also for muscles, kidneys, heart, and brain. Their physiologic production impacts the metabolism and transport through the epithelium, as well as epithelial cell renewal and differentiation. Moreover, SCFAs greatly influence the immune system, colonic functions, and carcinogenesis. Butyrate production, for example, improves gut barrier integrity and reduces local oxidative stress and inflammation (Macfarlane and Macfarlane, 2012). Recently, Kaiko et al. (2016) came with an interesting finding that butyrate levels are much lower at the intestinal crypt base than in the lumen. Differentiated enterocytes use butyrate as an energy source and thus reduce its concentration along the way to the lamina propria, where a low concentration of butyrate keeps the epithelial progenitors proliferating and stimulates tolerogenic immune response (Kaiko et al., 2016). The role of SCFAs in cancerogenesis is not fully understood but their concentration could be an important factor.

On the other hand, degradation and fermentation of dietary proteins, peptides, and amino acids by bacteria generates by-products, such as phenols, indoles, ammonia, amines, and hydrogen sulfite, all of which are to some extent harmful to the host, being co-carcinogens, mutagens, and cellular toxins (Macfarlane and Macfarlane, 2012). Moreover, hydrogen released as the end-product of fermentation is processed by methanogenic species of Archaea (e.g., *Methanobrevibacter*) to methane, which changes local conditions (redox potential and pH) and thus regulates biochemical pathways (Gill et al., 2006).

Fungal metabolic activity includes the digestion of polysaccharides and fat residuals from the diet and host residuals, leading to the synthesis of a variety of secondary metabolites, which can substantially influence the surrounding prokaryotic and eukaryotic cells. An investigation of the relationship between fungal diversity and diet revealed a positive correlation of *Candida* with diet rich in saccharides and a negative correlation of *Aspergillus* with SCFAs (Hoffmann et al., 2013). Thus, close relationships between bacterial and fungal metabolic requirements can help structure the microbial community in the gut. For instance, antimicrobial treatment can significantly disrupt the ecological balance of microbiota throughout the digestive tract. Antibiotics eradicate some sensitive bacteria and their niche can be subsequently invaded by other bacteria or fungi (Huffnagle and Noverr, 2013). Interestingly, a nested case-control study has shown that bacterial or fungal outgrowth after multiple penicillin treatments slightly increases the risk of CRC development (Boursi et al., 2015). And, last but not least, consumption of food-associated mycotoxins – secondary metabolites of fungi, has been linked to carcinogenesis throughout the digestive tract (De Ruyck et al., 2015). Several *in vitro* studies have shown that exposure to mycotoxins affects apoptosis, intestinal barrier integrity and mucus production and causes DNA damage, suggesting a possible role of mycotoxins in CRC development; reviewed by Maresca and Fantini (2010).

## Host–Microbiome Interaction and Disease Development

Host derived proteoglycans, forming the mucus layer, are an important part of the mucosal immune system. They protect the epithelium from an extensive contact with the microbiome and reduce the risk of microbial invasion. The oral cavity and esophagus harbor several layers of tight and largely inert squamous epithelium, whereas the remaining parts of the digestive tract are covered with a single layer of intensely active cells (Johansson et al., 2013). The structure of the mucus layers and types of mucin (MUC) vary widely along the digestive tract. The salivary glands in the oral cavity produce mainly MUC5B and MUC7, glands in the stomach and duodenum secrete gel-forming mucins MUC5AC and MUC6, and goblet cells in the gut specialize in MUC2 production (Khan et al., 1998; Wickstrom et al., 1998; Nordman et al., 2002). While in the small intestine, MUC2 forms a loose unattached mucus layer, in the colon it has two parts with different functions, an inner, attached layer and an outer, unattached one (Johansson et al., 2013). The inner layer, which is about 50–100 μm thick, is dense and impenetrable to most microbes, while the outer layer flows with the gut content. Mucus contains distinct products of epithelial cells, such as antimicrobial peptides and secretory IgA, which play an important role in the protection of gut mucosa against pathogen invasion or excessive inflammatory response to commensals (Johansson et al., 2011).

Interestingly, some members of the oral and gut microbiome can form a multilayer structure, composed of microbes and a polymeric matrix, termed a biofilm. Biofilm formation is one example of the mechanisms microorganisms use to evade antimicrobial defenses in the hostile environment of the host. Most biofilms are of polymicrobial nature and members of the biofilm community are distinct from the planktonic microbiota colonizing the mucosal surfaces throughout the body. Polymeric matrix formation and subsequent microbial colonization is the consequence of adhesion processes mediated by a wide spectrum of glycoproteins. Caries and periodontal disease are associated with biofilm formation by well-known periodontopathic bacteria. Biofilm in dental caries contains mainly streptococci, *L. acidophilus*, and *Actinomyces* and is secondarily colonized by anaerobic species, such as *F. nucleatum* and *P. gingivalis* (Chenicheri et al., 2017). In periodontal disease, early biofilm colonizers are mainly represented by streptococci and *Actinomyces*. Later on, more pathogenic bacteria such as *F. nucleatum*, *P. gingivalis*, *T. forsythia*, *T. denticola*, and *A. actinomycetemcomitans* appear (Socransky and Haffajee, 2005; Teles et al., 2013). The tendency of some microscopic fungi to form biofilms is also well-established in the literature. Recently, cooperation between *Candida* and oral commensal streptococci has been described as a significant factor in biofilm formation. Such cohabitation supports *Candida* growth and survival by providing it with an adhesive surface and the ability to invade tissue by promoting hyphae formation (Diaz et al., 2012).

Current research has confirmed the presence of polymicrobial biofilms on gut mucosa of CRC patients, suggesting their possible role in CRC pathogenesis. Even in healthy mucosa, biofilm formation is associated with oncogenic potential and might be used to predict susceptibility to cancer development (Dejea et al., 2014). These biofilms consist of periodontopathic bacteria – *F. nucleatum* and *P. gingivalis*, as well as oral commensals, such as *Peptostreptococcus*, *Prevotella*, and *Parvimonas*, and their metabolic products, which may contribute to CRC progression (Li et al., 2017). Microbial biofilms disrupt mucus layers, enabling potentially harmful microbes to attach to or even invade the mucosa and directly affect the epithelial cells by cytotoxic or genotoxic metabolites. Indeed, a recent study by Johnson et al. (2015) has provided evidence that the presence of biofilm increases polyamine metabolites in cancer tissues. Interestingly, fungal genera *Phoma* and *Candida* have been detected in higher quantities in adenoma biopsies (Luan et al., 2015). However, to date, the connection of these mixed-species biofilms with CRC has not been thoroughly studied.

When passing from the upper to the lower digestive tract, some previously mentioned bacteria change their oxygen requirements from facultative anaerobic to strict anaerobic, thereby switching to asaccharolytic and proteolytic metabolism (Eley and Cox, 2003). Microbial proteolytic enzymes break down the host’s extracellular matrix and soluble factors to get nutrients and invade the tissue. Periodontopathic bacteria produce a wide spectrum of enzymes, including collagenases, elastases, peptidases, etc. For instance, gingipains are cysteine proteases secreted by *P. gingivalis*, classified as either arginine (Rgp) or lysine (Kgp) specific (Potempa et al., 2003). They play a key role in biofilm formation, consequent host tissue destruction and vascular permeability induction (Kadowaki et al., 2000; Eley and Cox, 2003). Some of the *P. gingivalis* proteases can degrade immunologically active molecules, such as immunoglobulins, cytokines and components of the complement, and thus modulate the antibacterial immune response. Similarly, oral streptococci produce proteases which have been shown to cleave IgA1 (Kilian et al., 1988). Interestingly, 88% of the streptococci that initiate plaque formation on dental enamel possess IgA1 protease activity. Moreover, oral streptococci attack human immunoglobulin IgA1 not only by protease production but also by glycosidases (neuraminidase and beta-galactosidase). Oral streptococci thus cleave the alpha chains and also the carbohydrate moiety of IgA1. This finding suggests that the ability of streptococci to evade secretory immune mechanisms is one of the factors that enable them to colonize the oral cavity (Kilian et al., 1989).

Another important feature of the microbiota that protects the host against pathogens is resistance to outsider invasion. Microbiota presents a competitive barrier to pathogenic microbes by active struggle for existence, fighting for nutrients and niche occupation. Moreover, commensals express antimicrobial effector molecules (bacteriocins) that serve as an effective tool for community shaping by endogenous microbiota. The third mechanism is indirect through constitutive stimulation of the mucosal immune system by commensal microbes, which strengthens the mucosal barrier, thus reducing pathogen translocation (Robinson et al., 2010; Stecher and Hardt, 2011; Backhed et al., 2012).

Mucosal surface of the gut is in continuous contact with foreign compounds derived from diet as well as from commensal or pathogenic microorganisms. Therefore, maintaining balance between the inner and outer milieu is the hallmark of the whole mucosal immune system. Many different cell types and their products are involved in this complex dialog, including epithelial and immune cells, cells of supporting tissues, antimicrobial peptides, growth factors, cytokines, and other mediators. Various components of microbiota can differentially trigger cellular pathways that shape local as well as systemic immune response and physiological functions. Recognition of these microbe-associated molecular patterns (MAMPs) is one of the most important features of the mucosal immune system. Receptors facilitating this are known as pattern-recognition receptors and can be divided into several families, such as retinoic acid inducible gene I-like receptors, nod-like receptors, toll-like receptors (TLR), and lectin receptors. Proinflammatory processes that are mediated by MAMPs, such as lipopolysaccharide, polysaccharides, peptidoglycan, flagella, and microbial DNA/RNA, activate pattern-recognition receptors on various host cells. These cells elicit local pro-inflammatory response and/or drive the differentiation of adaptive immune response (Sartor, 2008; Underhill and Iliev, 2014). Therefore, a persistent inflammatory reaction of the host, constantly challenging the mucosal immune system, can lead to disease initiation (Tlaskalova-Hogenova et al., 2004). Appropriate immune response requires that recognition of commensals on the apical side of the epithelium induces tolerance, while recognition of pathogens on the basolateral membrane or inside the cell induces inflammation. Moreover, TLRs are important for stimulation of gut epithelium growth and barrier integrity as well as production of mucus, secretory IgA, antimicrobial peptides, and chemokines (Abreu, 2010). Generally, the expression of *TLR*s in the epithelium is low in the steady state but increases during inflammation. Indeed, recent studies suggested the association of *TLR* polymorphisms with the progression of IBD into CRC, as *TLR*s expression is changed during gut inflammation (Bank et al., 2015).

Chronic inflammation leads to massive accumulation of activated immune cells and their mediators (cytokines and chemokines), residues of damaged cells, and large amounts of oxygen and nitrogen reactive species. Isolated dysplastic cells are further modified by the local microenvironment, as pro-inflammatory cells and cytokines promote the progression of dysplasia into carcinoma. During chronic inflammation, innate immune cells produce excessive quantities of reactive oxygen and nitrogen species that cause DNA and cellular damage. Pattern-recognition receptors signalization in the milieu of chronic inflammation activates MyD88-dependent pathways that promote pro-inflammatory cytokine release and subsequent tumor progression (Tlaskalova-Hogenova et al., 2014).

## Conclusion

**FIGURE 2:**
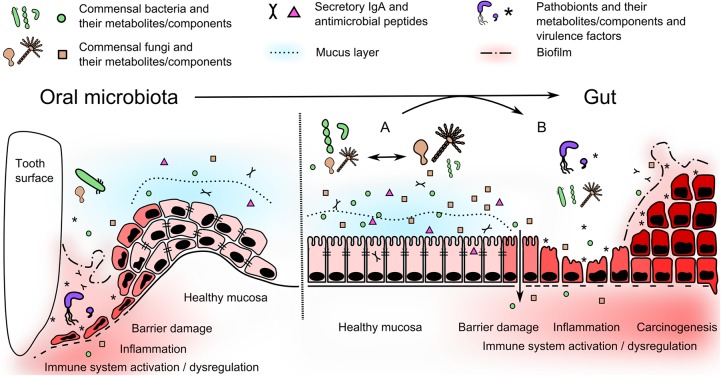
Oral microbiota moves to the gut and alter local microenvironment. In oral cavity, periodontal pathogens use several mechanisms, such as adhesion, biofilm formation and protease activity (see **Table 1**), to disrupt the barrier and trigger the inflammatory changes in periodontium. In the gut, healthy stable microbiome can undergo temporary dynamic perturbations promoting growth of bacterial or fungal part of the microbiome **(A)**. These shifts are mostly reversible, do not disrupt mucosal barrier function, nor induce proinflammatory immune response. Nevertheless, microbes associated with periodontal disease can further disseminate into the gut becoming a part of altered gut microbiome with pathogenic potential. They can use similar mechanisms as in oral cavity and produce virulence and growth-promoting factors or substitute missing beneficial commensals. This can affect the stability of resident microbiome and promote their selective outgrowth leading to a dysbiosis **(B)**. Subsequent adhesion and biofilm formation, proteolytic activity or accumulation of harmful metabolites and soluble components can damage the integrity of gut barrier. These pathogenic mechanisms, together with dysregulated host immune response, can lead to inflammation and/or colorectal cancer initiation.

There are two fundamental links between microbes and diseases. The first involves the host’s recognition and immune response mechanisms and the second involves the microbiota itself, its presence and metabolic activity. Impaired barrier function, inadequate activation of the innate immune system and dysregulation of the appropriate mucosal immune response to gut microbiota (tolerance) are the primary elements of disease development. Low microbiome diversity seems to be a common feature in the pathogenesis of diseases of the digestive tract, suggesting that high microbial richness, both bacterial and fungal, is crucial for physiological homeostasis. Normally, a dynamic balance is maintained, where reduction of the bacterial community leads to outgrowth of the fungal one and vice versa. Disruption of this balance is associated with outgrowth of certain pathobionts at the expense of a complex community and may lead to disease initiation (**Figure 2**). Established dysbiosis is supported by local proinflammatory and tumor microenvironment which creates distinct nutritional conditions. A growing body of literature dealing with microbiome metabolic activities gradually unravels the complex host–microbe interactions. Nevertheless, collecting data about these interactions at the time of disease development is especially challenging in humans. In this respect, we still have to rely on retrospective clinical studies and animal models to generate helpful insights. Elucidation of the underlying mechanisms involving the interactions of the immune system with the microbiome, pathogens, or pathobionts can lead to the development of early screening and preventive interventions and, ultimately, reduce the risk of severe colon cancer.

## Author Contributions

KK, ZJZ, and HT-H wrote the manuscript and approved its final version.

## Conflict of Interest Statement

The authors declare that the research was conducted in the absence of any commercial or financial relationships that could be construed as a potential conflict of interest.

## References

[B1] Aagaard K. Ma J. Antony K. M. Ganu R. Petrosino J. Versalovic J. (2014). The placenta harbors a unique microbiome. *Sci. Transl. Med.* 6:237ra65. 10.1126/scitranslmed.3008599 24848255 PMC4929217

[B2] Aas J. A. Paster B. J. Stokes L. N. Olsen I. Dewhirst F. E. (2005). Defining the normal bacterial flora of the oral cavity. *J. Clin. Microbiol.* 43 5721–5732. 10.1128/Jcm.43.11.5721-5732.2005 16272510 PMC1287824

[B3] Abed J. Emgard J. E. M. Zamir G. Faroja M. Almogy G. Grenov A. (2016). Fap2 mediates *Fusobacterium nucleatum* colorectal adenocarcinoma enrichment by binding to tumor-expressed Gal-GalNAc. *Cell Host Microbe* 20 215–225. 10.1016/j.chom.2016.07.006 27512904 PMC5465824

[B4] Abreu M. T. (2010). Toll-like receptor signalling in the intestinal epithelium: how bacterial recognition shapes intestinal function. *Nat. Rev. Immunol.* 10 131–144. 10.1038/nri2707 20098461

[B5] Adlerberth I. Wold A. E. (2009). Establishment of the gut microbiota in Western infants. *Acta Paediatr.* 98 229–238. 10.1111/j.1651-2227.2008.01060.x 19143664

[B6] Amitay E. L. Werner S. Vital M. Pieper D. H. Hofler D. Gierse I. J. (2017). Fusobacterium and colorectal cancer: causal factor or passenger? Results from a large colorectal cancer screening study. *Carcinogenesis* 38 781–788. 10.1093/carcin/bgx053 28582482

[B7] Arthur J. C. Jobin C. (2011). The struggle within: microbial influences on colorectal cancer. *Inflamm. Bowel Dis.* 17 396–409. 10.1002/ibd.21354 20848537 PMC3376405

[B8] Arthur J. C. Perez-Chanona E. Muhlbauer M. Tomkovich S. Uronis J. M. Fan T. J. (2012). Intestinal inflammation targets cancer-inducing activity of the microbiota. *Science* 338 120–123. 10.1126/science.1224820 22903521 PMC3645302

[B9] Arumugam M. Raes J. Pelletier E. Le Paslier D. Yamada T. Mende D. R. (2011). Enterotypes of the human gut microbiome. *Nature* 473 174–180. 10.1038/nature09944 21508958 PMC3728647

[B10] Backhed F. Fraser C. M. Ringel Y. Sanders M. E. Sartor R. B. Sherman P. M. (2012). Defining a healthy human gut microbiome: current concepts, future directions, and clinical applications. *Cell Host Microbe* 12 611–622. 10.1016/j.chom.2012.10.012 23159051

[B11] Bank S. Andersen P. S. Burisch J. Pedersen N. Roug S. Galsgaard J. (2015). Polymorphisms in the toll-like receptor and the IL-23/IL-17 pathways were associated with susceptibility to inflammatory bowel disease in a danish cohort. *PLoS One* 10:e0145302. 10.1371/journal.pone.0145302 26698117 PMC4689491

[B12] Bankvall M. Sjoberg F. Gale G. Wold A. Jontell M. Ostman S. (2014). The oral microbiota of patients with recurrent aphthous stomatitis. *J. Oral Microbiol.* 6:25739. 10.3402/Jom.V6.25739 25626771 PMC4221501

[B13] Bartova J. Sommerova P. Lyuya-Mi Y. Mysak J. Prochazkova J. Duskova J. (2014). Periodontitis as a risk factor of atherosclerosis. *J. Immunol. Res.* 2014:636893. 10.1155/2014/636893 24741613 PMC3987959

[B14] Blazkova H. Krejcikova K. Moudry P. Frisan T. Hodny Z. Bartek J. (2010). Bacterial intoxication evokes cellular senescence with persistent DNA damage and cytokine signalling. *J. Cell Mol. Med.* 14 357–367. 10.1111/j.1582-4934.2009.00862.x 19650831 PMC3837606

[B15] Boursi B. Haynes K. Mamtani R. Yang Y. X. (2015). Impact of antibiotic exposure on the risk of colorectal cancer. *Pharmacoepidemiol. Drug Saf.* 24 534–542. 10.1002/pds.3765 25808540

[B16] Canabarro A. Valle C. Farias M. R. Santos F. B. Lazera M. Wanke B. (2013). Association of subgingival colonization of *Candida albicans* and other yeasts with severity of chronic periodontitis. *J. Periodontal Res.* 48 428–432. 10.1111/jre.12022 23137301

[B17] Carlsson J. Larsen J. T. Edlund M. B. (1993). *Peptostreptococcus micros* has a uniquely high capacity to form hydrogen sulfide from glutathione. *Oral Microbiol. Immunol.* 8 42–45. 10.1111/j.1399-302X.1993.tb00541.x 8510983

[B18] Casarin R. C. Saito D. Santos V. R. Pimentel S. P. Duarte P. M. Casati M. Z. (2012). Detection of *Mogibacterium timidum* in subgingival biofilm of aggressive and non-diabetic and diabetic chronic periodontitis patients. *Braz. J. Microbiol.* 43 931–937. 10.1590/S1517-838220120003000012 24031909 PMC3768883

[B19] Castellarin M. Warren R. L. Freeman J. D. Dreolini L. Krzywinski M. Strauss J. (2012). *Fusobacterium nucleatum* infection is prevalent in human colorectal carcinoma. *Genome Res.* 22 299–306. 10.1101/gr.126516.111 22009989 PMC3266037

[B20] Chehoud C. Albenberg L. G. Judge C. Hoffmann C. Grunberg S. Bittinger K. (2015). Fungal signature in the gut microbiota of pediatric patients with inflammatory bowel disease. *Inflamm. Bowel Dis.* 21 1948–1956. 10.1097/MIB.0000000000000454 26083617 PMC4509842

[B21] Chenicheri S. Usha R. Ramachandran R. Thomas V. Wood A. (2017). Insight into oral biofilm: primary, secondary and residual caries and phyto-challenged solutions. *Open Dent J.* 11 312–333. 10.2174/1874210601711010312 28839480 PMC5543615

[B22] Claesson R. Edlund M. B. Persson S. Carlsson J. (1990). Production of volatile sulfur compounds by various Fusobacterium species. *Oral Microbiol. Immunol.* 5 137–142. 10.1111/j.1399-302X.1990.tb00411.x 2080068

[B23] Cobb C. M. Kelly P. J. Williams K. B. Babbar S. Angolkar M. Derman R. J. (2017). The oral microbiome and adverse pregnancy outcomes. *Int. J. Womens Health* 9 551–559. 10.2147/Ijwh.S142730 28848365 PMC5557618

[B24] Costalonga M. Herzberg M. C. (2014). The oral microbiome and the immunobiology of periodontal disease and caries. *Immunol. Lett.* 162 22–38. 10.1016/j.imlet.2014.08.017 25447398 PMC4346134

[B25] da Silva E. S. Feres M. Figueiredo L. C. Shibli J. A. Ramiro F. S. Faveri M. (2014). Microbiological diversity of peri-implantitis biofilm by Sanger sequencing. *Clin. Oral Implants Res.* 25 1192–1199. 10.1111/clr.12231 23845046

[B26] Darveau R. P. Hajishengallis G. Curtis M. A. (2012). *Porphyromonas gingivalis* as a potential community activist for disease. *J. Dent. Res.* 91 816–820. 10.1177/0022034512453589 22772362 PMC3420389

[B27] De Ruyck K. De Boevre M. Huybrechts I. De Saeger S. (2015). Dietary mycotoxins, co-exposure, and carcinogenesis in humans: short review. *Mutat. Res. Rev. Mutat. Res.* 766 32–41. 10.1016/j.mrrev.2015.07.003 26596546

[B28] Dejea C. M. Wick E. C. Hechenbleikner E. M. White J. R. Mark Welch J. L. Rossetti B. J. (2014). Microbiota organization is a distinct feature of proximal colorectal cancers. *Proc. Natl. Acad. Sci. U.S.A.* 111 18321–18326. 10.1073/pnas.1406199111 25489084 PMC4280621

[B29] Diaz P. I. Xie Z. H. Sobue T. Thompson A. Biyikoglu B. Ricker A. (2012). Synergistic interaction between *Candida albicans* and commensal oral streptococci in a novel *in vitro* mucosal model. *Infect. Immun.* 80 620–632. 10.1128/Iai.05896-11 22104105 PMC3264323

[B30] Dupuy A. K. David M. S. Li L. Heider T. N. Peterson J. D. Montano E. A. (2014). Redefining the human oral mycobiome with improved practices in amplicon-based taxonomy: discovery of malassezia as a prominent commensal. *PLoS One* 9:e90899. 10.1371/journal.pone.0090899 24614173 PMC3948697

[B31] Egeberg A. Mallbris L. Gislason G. Hansen P. R. Mrowietz U. (2017). Risk of periodontitis in patients with psoriasis and psoriatic arthritis. *J. Eur. Acad. Dermatol. Venereol.* 31 288–293. 10.1111/jdv.13814 27439545

[B32] El-Aouar R. A. Nicolas A. Castro T. L. D. Deplanche M. Azevedo V. A. D. Goossens P. L. (2017). Heterogeneous family of cyclomodulins: smart weapons that allow bacteria to hijack the eukaryotic cell cycle and promote infections. *Front. Cell Infect. Microbiol.* 7:208 10.3389/Fcimb.2017.00364 PMC5440457 28589102

[B33] Eley B. M. Cox S. W. (2003). Proteolytic and hydrolytic enzymes from putative periodontal pathogens: characterization, molecular genetics, effects on host defenses and tissues and detection in gingival crevice fluid. *Periodontol* 31 105–124. 10.1034/j.1600-0757.2003.03107.x 12656998

[B34] Fan X. Alekseyenko A. V. Wu J. Jacobs E. J. Gapstur S. M. Purdue M. P. (2016). Human oral microbiome and prospective risk for pancreatic cancer: a population based, nested case control study. *Cancer Res.* 76:4350. 10.1158/1538-7445.AM2016-4350 27742762 PMC5607064

[B35] Ferlay J. Soerjomataram I. Dikshit R. Eser S. Mathers C. Rebelo M. (2015). Cancer incidence and mortality worldwide: sources, methods and major patterns in GLOBOCAN 2012. *Int. J. Cancer* 136 E359–E386. 10.1002/ijc.29210 25220842

[B36] Flanagan L. Schmid J. Ebert M. Soucek P. Kunicka T. Liska V. (2014). *Fusobacterium nucleatum* associates with stages of colorectal neoplasia development, colorectal cancer and disease outcome. *Eur. J. Clin. Microbiol. Infect. Dis.* 33 1381–1390. 10.1007/s10096-014-2081-3 24599709

[B37] Flemer B. Lynch D. B. Brown J. M. Jeffery I. B. Ryan F. J. Claesson M. J. (2017). Tumour-associated and non-tumour-associated microbiota in colorectal cancer. *Gut* 66 633–643. 10.1136/gutjnl-2015-309595 26992426 PMC5529966

[B38] Ford P. J. Gemmell E. Chan A. Carter C. L. Walker P. J. Bird P. S. (2006). Inflammation, heat shock proteins and periodontal pathogens in atherosclerosis: an immunohistologic study. *Oral Microbiol. Immunol.* 21 206–211. 10.1111/j.1399-302X.2006.00276.x 16842503

[B39] Ford P. J. Gemmell E. Hamlet S. M. Hasan A. Walker P. J. West M. J. (2005). Cross-reactivity of GroEL antibodies with human heat shock protein 60 and quantification of pathogens in atherosclerosis. *Oral Microbiol. Immunol.* 20 296–302. 10.1111/j.1399-302X.2005.00230.x 16101965

[B40] Gagniere J. Raisch J. Veziant J. Barnich N. Bonnet R. Buc E. (2016). Gut microbiota imbalance and colorectal cancer. *World J. Gastroenterol.* 22 501–518. 10.3748/wjg.v22.i2.501 26811603 PMC4716055

[B41] Ghannoum M. A. Jurevic R. J. Mukherjee P. K. Cui F. Sikaroodi M. Naqvi A. (2010). Characterization of the oral fungal microbiome (Mycobiome) in healthy individuals. *PLoS Pathog.* 6:e1000713. 10.1371/journal.ppat.1000713 20072605 PMC2795202

[B42] Gill S. R. Pop M. DeBoy R. T. Eckburg P. B. Turnbaugh P. J. Samuel B. S. (2006). Metagenomic analysis of the human distal gut microbiome. *Science* 312 1355–1359. 10.1126/science.1124234 16741115 PMC3027896

[B43] Gomes C. C. Guimarães L. S. Pinto L. C. C. Camargo G. A. D. C. G. Valente M. I. B. Sarquis M. I. M. (2017). Investigations of the prevalence and virulence of *Candida albicans* in periodontal and endodontic lesions in diabetic and normoglycemic patients. *J. Appl. Oral Sci.* 25 274–281. 10.1590/1678-7757-2016-0432 28678946 PMC5482250

[B44] Greenwald R. A. Kirkwood K. (1999). Adult periodontitis as a model for rheumatoid arthritis (with emphasis on treatment strategies). *J. Rheumatol.* 26 1650–1653. 10451056

[B45] Hajishengallis G. Korostoff J. M. (2017). Revisiting the page & schroeder model: the good, the bad and the unknowns in the periodontal host response 40 years later. *Periodontol* 75 116–151. 10.1111/prd.12181 28758305 PMC5539911

[B46] Hale V. L. Chen J. Johnson S. Harrington S. C. Yab T. C. Smyrk T. C. (2017). Shifts in the fecal microbiota associated with adenomatous polyps. *Cancer Epidemiol. Biomarkers Prev.* 26 85–94. 10.1158/1055-9965.EPI-16-0337 27672054 PMC5225053

[B47] Han Y. W. (2015). *Fusobacterium nucleatum*: a commensal-turned pathogen. *Curr. Opin. Microbiol.* 23 141–147. 10.1016/j.mib.2014.11.013 25576662 PMC4323942

[B48] He J. Z. Li Y. Cao Y. P. Xue J. Zhou X. D. (2015). The oral microbiome diversity and its relation to human diseases. *Folia Microbiol.* 60 69–80. 10.1007/s12223-014-0342-2 25147055

[B49] Heijl L. Wennstrom J. Lindhe J. Socransky S. S. (1980). Periodontal-disease in gnotobiotic-rats. *J. Periodontal Res.* 15 405–419. 10.1111/j.1600-0765.1980.tb00298.x 6449576

[B50] Hoffmann C. Dollive S. Grunberg S. Chen J. Li H. Wu G. D. (2013). Archaea and fungi of the human gut microbiome: correlations with diet and bacterial residents. *PLoS One* 8:e66019. 10.1371/journal.pone.0066019 23799070 PMC3684604

[B51] Huffnagle G. B. Noverr M. C. (2013). The emerging world of the fungal microbiome. *Trends Microbiol.* 21 334–341. 10.1016/j.tim.2013.04.002 23685069 PMC3708484

[B52] Human Microbiome Project Consortium (2012). Structure, function and diversity of the healthy human microbiome. *Nature* 486 207–214. 10.1038/nature11234 22699609 PMC3564958

[B53] Huse S. M. Ye Y. Z. Zhou Y. J. Fodor A. A. (2012). A core human microbiome as viewed through 16S rRNA sequence clusters. *PLoS One* 7:e34242. 10.1371/journal.pone.0034242 22719824 PMC3374614

[B54] Huycke M. M. Abrams V. Moore D. R. (2002). Enterococcus faecalis produces extracellular superoxide and hydrogen peroxide that damages colonic epithelial cell DNA. *Carcinogenesis* 23 529–536. 10.1093/carcin/23.3.529 11895869

[B55] Inaba H. Sugita H. Kuboniwa M. Iwai S. Hamada M. Noda T. (2013). *Porphyromonas gingivalis* promotes invasion of oral squamous cell carcinoma through induction of proMMP9 and its activation. *Cell Microbiol.* 16 131–145. 10.1111/cmi.12211 23991831 PMC3939075

[B56] Ivanyi L. Newman H. N. Marsh P. D. (1991). T-cell proliferative responses to molecular fractions of periodontopathic bacteria. *Clin. Exp. Immunol.* 83 108–111. 10.1111/j.1365-2249.1991.tb05597.x 1988218 PMC1535464

[B57] Johansson M. E. Larsson J. M. Hansson G. C. (2011). The two mucus layers of colon are organized by the MUC2 mucin, whereas the outer layer is a legislator of host-microbial interactions. *Proc. Natl. Acad. Sci. U.S.A.* 108 4659–4665. 10.1073/pnas.1006451107 20615996 PMC3063600

[B58] Johansson M. E. Sjovall H. Hansson G. C. (2013). The gastrointestinal mucus system in health and disease. *Nat. Rev. Gastroenterol. Hepatol.* 10 352–361. 10.1038/nrgastro.2013.35 23478383 PMC3758667

[B59] Johnson C. H. Dejea C. M. Edler D. Hoang L. T. Santidrian A. F. Felding B. H. (2015). Metabolism links bacterial biofilms and colon carcinogenesis. *Cell Metab.* 21 891–897. 10.1016/j.cmet.2015.04.011 25959674 PMC4456201

[B60] Kadowaki T. Nakayama K. Okamoto K. Abe N. Baba A. Shi Y. X. (2000). *Porphyromonas gingivalis* proteinases as virulence determinants in progression of periodontal diseases. *J. Biochem.* 128 153–159. 10.1093/oxfordjournals.jbchem.a022735 10920248

[B61] Kaiko G. E. Ryu S. H. Koues O. I. Collins P. L. Solnica-Krezel L. Pearce E. J. (2016). The colonic crypt protects stem cells from microbiota-derived metabolites. *Cell* 165 1708–1720. 10.1016/j.cell.2016.10.034 27264604 PMC5026192

[B62] Kasser U. R. Gleissner C. Dehne F. Michel A. Willershausen-Zonnchen B. Bolten W. W. (1997). Risk for periodontal disease in patients with longstanding rheumatoid arthritis. *Arthritis Rheum.* 40 2248–2251. 10.1002/art.1780401221 9416864

[B63] Khan S. H. Aguirre A. Bobek L. A. (1998). In-situ hybridization localized MUC7 mucin gene expression to the mucous acinar cells of human and MUC7-transgenic mouse salivary glands. *Glycoconj. J.* 15 1125–1132. 10.1023/A:1006955604501 10372967

[B64] Kilian M. Mestecky J. Russell M. W. (1988). Defense mechanisms involving Fc-dependent functions of immunoglobulin A and their subversion by bacterial immunoglobulin A proteases. *Microbiol. Rev.* 52 296–303. 3045518 10.1128/mr.52.2.296-303.1988 PMC373140

[B65] Kilian M. Reinholdt J. Nyvad B. Frandsen E. V. Mikkelsen L. (1989). IgA1 proteases of oral streptococci: ecological aspects. *Immunol. Invest.* 18 161–170. 10.3109/08820138909112235 2659509

[B66] Kim Y. J. Choi Y. S. Baek K. J. Yoon S. H. Park H. K. Choi Y. (2016). Mucosal and salivary microbiota associated with recurrent aphthous stomatitis. *BMC Microbiol.* 16:57. 10.1186/s12866-016-0673-z 27036492 PMC4818471

[B67] Kinane D. F. Stathopoulou P. G. Papapanou P. N. (2017). Periodontal diseases. *Nat. Rev. Dis. Primers* 3:17038. 10.1038/Nrdp.2017.38 28805207

[B68] Klimesova K. Kverka M. Zakostelska Z. Hudcovic T. Hrncir T. Stepankova R. (2013). Altered gut microbiota promotes colitis-associated cancer in IL-1 receptor-associated kinase M-deficient mice. *Inflamm. Bowel Dis.* 19 1266–1277. 10.1097/MIB.0b013e318281330a 23567778 PMC3744230

[B69] Kostic A. D. Chun E. Y. Robertson L. Glickman J. N. Gallini C. A. Michaud M. (2013). *Fusobacterium nucleatum* potentiates intestinal tumorigenesis and modulates the tumor-immune microenvironment. *Cell Host Microbe* 14 207–215. 10.1016/j.chom.2013.07.007 23954159 PMC3772512

[B70] Kostic A. D. Gevers D. Pedamallu C. S. Michaud M. Duke F. Earl A. M. (2012). Genomic analysis identifies association of Fusobacterium with colorectal carcinoma. *Genome Res.* 22 292–298. 10.1101/gr.126573.111 22009990 PMC3266036

[B71] Li S. Konstantinov S. R. Smits R. Peppelenbosch M. P. (2017). Bacterial biofilms in colorectal cancer initiation and progression. *Trends Mol. Med.* 23 18–30. 10.1016/j.molmed.2016.11.004 27986421

[B72] Liang Q. Chiu J. Chen Y. Huang Y. Higashimori A. Fang J. (2017). Fecal bacteria act as novel biomarkers for noninvasive diagnosis of colorectal cancer. *Clin. Cancer Res.* 23 2061–2070. 10.1158/1078-0432.CCR-16-1599 27697996

[B73] Liguori G. Lamas B. Richard M. L. Brandi G. da Costa G. Hoffmann T. W. (2016). Fungal dysbiosis in mucosa-associated microbiota of crohn’s disease patients. *J. Crohns Colitis* 10 296–305. 10.1093/ecco-jcc/jjv209 26574491 PMC4957473

[B74] Louis P. Young P. Holtrop G. Flint H. J. (2010). Diversity of human colonic butyrate-producing bacteria revealed by analysis of the butyryl-CoA: acetate CoA-transferase gene. *Environ. Microbiol.* 12 304–314. 10.1111/j.1462-2920.2009.02066.x 19807780

[B75] Lu Y. Chen J. Zheng J. Hu G. Wang J. Huang C. (2016). Mucosal adherent bacterial dysbiosis in patients with colorectal adenomas. *Sci. Rep.* 6:26337. 10.1038/srep26337 27194068 PMC4872055

[B76] Luan C. Xie L. Yang X. Miao H. Lv N. Zhang R. (2015). Dysbiosis of fungal microbiota in the intestinal mucosa of patients with colorectal adenomas. *Sci. Rep.* 5:7980. 10.1038/srep07980 25613490 PMC4648387

[B77] Macfarlane G. T. Macfarlane S. (2012). Bacteria, colonic fermentation, and gastrointestinal health. *J. AOAC Int.* 95 50–60. 10.5740/jaoacint.SGE_Macfarlane 22468341

[B78] Maffei V. J. Kim S. Blanchard E. Luo M. Jazwinski S. M. Taylor C. M. (2017). Biological aging and the human gut microbiota. *J. Gerontol. A Biol. Sci. Med. Sci.* 72 1474–1482. 10.1093/gerona/glx042 28444190 PMC5861892

[B79] Mager D. L. Ximenez-Fyvie L. A. Haffajee A. D. Socransky S. S. (2003). Distribution of selected bacterial species on intraoral surfaces. *J. Clin. Periodontol.* 30 644–654. 10.1034/j.1600-051X.2003.00376.x 12834503

[B80] Mao S. Park Y. Hasegawa Y. Tribble G. D. James C. E. Handfield M. (2007). Intrinsic apoptotic pathways of gingival epithelial cells modulated by *Porphyromonas gingivalis*. *Cell Microbiol.* 9 1997–2007. 10.1111/j.1462-5822.2007.00931.x 17419719 PMC2886729

[B81] Marchesan J. Jiao Y. Schaff R. A. Hao J. Morelli T. Kinney J. S. (2016). TLR4, NOD1 and NOD2 mediate immune recognition of putative newly identified periodontal pathogens. *Mol. Oral Microbiol.* 31 243–258. 10.1111/omi.12116 26177212 PMC4713362

[B82] Marcotte H. Lavoie M. C. (1998). Oral microbial ecology and the role of salivary immunoglobulin A. *Microbiol. Mol. Biol. Rev.* 62 71–109. 9529888 10.1128/mmbr.62.1.71-109.1998 PMC98907

[B83] Maresca M. Fantini J. (2010). Some food-associated mycotoxins as potential risk factors in humans predisposed to chronic intestinal inflammatory diseases. *Toxicon* 56 282–294. 10.1016/j.toxicon.2010.04.016 20466014

[B84] Mehta R. S. Nishihara R. Cao Y. Song M. Mima K. Qian Z. R. (2017). Association of dietary patterns with risk of colorectal cancer subtypes classified by *Fusobacterium nucleatum* in tumor tissue. *JAMA Oncol.* 3 921–927. 10.1001/jamaoncol.2016.6374 28125762 PMC5502000

[B85] Miraglia A. G. Travaglione S. Meschini S. Falzano L. Matarrese P. Quaranta M. G. (2007). Cytotoxic necrotizing factor 1 prevents apoptosis via the Akt/IkappaB kinase pathway: role of nuclear factor-kappaB and Bcl-2. *Mol. Biol. Cell* 18 2735–2744. 10.1091/mbc.E06-10-0910 17507655 PMC1924812

[B86] Momen-Heravi F. Babic A. Tworoger S. S. Zhang L. Wu K. Smith-Warner S. A. (2017). Periodontal disease, tooth loss and colorectal cancer risk: results from the Nurses’ Health Study. *Int. J. Cancer* 140 646–652. 10.1002/ijc.30486 27778343 PMC5159274

[B87] Mukherjee P. K. Chandra J. Retuerto M. Sikaroodi M. Brown R. E. Jurevic R. (2014). Oral mycobiome analysis of HIV-infected patients: identification of Pichia as an antagonist of opportunistic fungi. *PLoS Pathog.* 10:e1003996. 10.1371/journal.ppat.1003996 24626467 PMC3953492

[B88] Nagano Y. Elborn J. S. Miller B. C. Walker J. M. Goldsmith C. E. Rendall J. (2010). Comparison of techniques to examine the diversity of fungi in adult patients with cystic fibrosis. *Med. Mycol.* 48 166–176. 10.3109/13693780903127506 19672783

[B89] Nakatsu G. Li X. C. Zhou H. K. Sheng J. Q. Wong S. H. Wu W. K. K. (2015). Gut mucosal microbiome across stages of colorectal carcinogenesis. *Nat. Commun.* 6:8727. 10.1038/Ncomms9727 26515465 PMC4640069

[B90] Nordman H. Davies J. R. Lindell G. de Bolos C. Real F. Carlstedt I. (2002). Gastric MUC5AC and MUC6 are large oligomeric mucins that differ in size, glycosylation and tissue distribution. *Biochem. J.* 364 191–200. 10.1042/bj3640191 11988092 PMC1222561

[B91] Nougayrede J. P. Homburg S. Taieb F. Boury M. Brzuszkiewicz E. Gottschalk G. (2006). *Escherichia coli* induces DNA double-strand breaks in eukaryotic cells. *Science* 313 848–851. 10.1126/science.1127059 16902142

[B92] Papadopoulos G. Weinberg E. O. Massari P. Gibson F. C. III Wetzler L. M. Morgan E. F. (2013). Macrophage-specific TLR2 signaling mediates pathogen-induced TNF-dependent inflammatory oral bone loss. *J. Immunol.* 190 1148–1157. 10.4049/jimmunol.1202511 23264656 PMC3549226

[B93] Park H. E. Kim J. H. Cho N. Y. Lee H. S. Kang G. H. (2017). Intratumoral *Fusobacterium nucleatum* abundance correlates with macrophage infiltration and CDKN2A methylation in microsatellite-unstable colorectal carcinoma. *Virchows Arch.* 471 329–336. 10.1007/s00428-017-2171-6 28597080

[B94] Persson S. Edlund M. B. Claesson R. Carlsson J. (1990). The formation of hydrogen sulfide and methyl mercaptan by oral bacteria. *Oral Microbiol. Immunol.* 5 195–201. 10.1111/j.1399-302X.1990.tb00645.x 2082242

[B95] Peters B. A. Dominianni C. Shapiro J. A. Church T. R. Wu J. Miller G. (2016). The gut microbiota in conventional and serrated precursors of colorectal cancer. *Microbiome* 4:69. 10.1186/s40168-016-0218-6 28038683 PMC5203720

[B96] Peters B. A. Wu J. Hayes R. B. Ahn J. (2017). The oral fungal mycobiome: characteristics and relation to periodontitis in a pilot study. *BMC Microbiol.* 17:157. 10.1186/s12866-017-1064-9 28701186 PMC5508751

[B97] Pitts N. B. Zero D. T. Marsh P. D. Ekstrand K. Weintraub J. A. Ramos-Gomez F. (2017). Dental caries. *Nat. Rev. Dis. Primers* 3:17030. 10.1038/nrdp.2017.30 28540937

[B98] Potempa J. Sroka A. Imamura T. Travis J. (2003). Gingipains, the major cysteine proteinases and virulence factors of *Porphyromonas gingivalis*: structure, function and assembly of multidomain protein complexes. *Curr. Protein Pept. Sci.* 4 397–407. 10.2174/1389203033487036 14683426

[B99] Potgieter M. Bester J. Kell D. B. Pretorius E. (2015). The dormant blood microbiome in chronic, inflammatory diseases. *FEMS Microbiol. Rev.* 39 567–591. 10.1093/femsre/fuv013 25940667 PMC4487407

[B100] Proctor D. M. Relman D. A. (2017). The landscape ecology and microbiota of the human nose, mouth, and throat. *Cell Host Microbe* 21 421–432. 10.1016/j.chom.2017.03.011 28407480 PMC5538306

[B101] Qin J. Li R. Raes J. Arumugam M. Burgdorf K. S. Manichanh C. (2010). A human gut microbial gene catalogue established by metagenomic sequencing. *Nature* 464 59–65. 10.1038/nature08821 20203603 PMC3779803

[B102] Raisch J. Buc E. Bonnet M. Sauvanet P. Vazeille E. de Vallee A. (2014). Colon cancer-associated B2 *Escherichia coli* colonize gut mucosa and promote cell proliferation. *World J. Gastroenterol.* 20 6560–6572. 10.3748/wjg.v20.i21.6560 24914378 PMC4047342

[B103] Reddy B. S. Narisawa T. Maronpot R. Weisburger J. H. Wynder E. L. (1975). Animal models for the study of dietary factors and cancer of the large bowel. *Cancer Res.* 35 3421–3426. 1192409

[B104] Roberts F. A. Darveau R. P. (2015). Microbial protection and virulence in periodontal tissue as a function of polymicrobial communities: symbiosis and dysbiosis. *Periodontol* 69 18–27. 10.1111/prd.12087 26252399 PMC4530467

[B105] Robinson C. J. Bohannan B. J. M. Young V. B. (2010). From structure to function: the ecology of host-associated microbial communities. *Microbiol. Mol. Biol. R* 74 453–476. 10.1128/Mmbr.00014-10 20805407 PMC2937523

[B106] Rubinstein M. R. Wang X. Liu W. Hao Y. Cai G. Han Y. W. (2013). *Fusobacterium nucleatum* promotes colorectal carcinogenesis by modulating E-cadherin/beta-catenin signaling via its FadA adhesin. *Cell Host Microbe* 14 195–206. 10.1016/j.chom.2013.07.012 23954158 PMC3770529

[B107] Sartor R. B. (2008). Microbial influences in inflammatory bowel diseases. *Gastroenterology* 134 577–594. 10.1053/j.gastro.2007.11.059 18242222

[B108] Saunders C. W. Scheynius A. Heitman J. (2012). Malassezia fungi are specialized to live on skin and associated with dandruff, eczema, and other skin diseases. *PLoS Pathog.* 8:e1002701. 10.1371/journal.ppat.1002701 22737067 PMC3380954

[B109] Segata N. Haake S. K. Mannon P. Lemon K. P. Waldron L. Gevers D. (2012). Composition of the adult digestive tract bacterial microbiome based on seven mouth surfaces, tonsils, throat and stool samples. *Genome Biol.* 13:R42. 10.1186/gb-2012-13-6-r42 22698087 PMC3446314

[B110] Shen X. J. Rawls J. F. Randall T. Burcal L. Mpande C. N. Jenkins N. (2010). Molecular characterization of mucosal adherent bacteria and associations with colorectal adenomas. *Gut Microbes* 1 138–147. 10.4161/gmic.1.3.12360 20740058 PMC2927011

[B111] Slots J. (1976). Predominant cultivable organisms in juvenile periodontitis. *Scand. J. Dent. Res.* 84 1–10. 10.1111/j.1600-0722.1976.tb00454.x 1061986

[B112] Socransky S. S. Haffajee A. D. (2002). Dental biofilms: difficult therapeutic targets. *Periodontol* 28 12–55. 10.1034/j.1600-0757.2002.280102.x 12013340

[B113] Socransky S. S. Haffajee A. D. (2005). Periodontal microbial ecology. *Periodontol* 38 135–187. 10.1111/j.1600-0757.2005.00107.x 15853940

[B114] Socransky S. S. Haffajee A. D. Cugini M. A. Smith C. Kent R. L. (1998). Microbial complexes in subgingival plaque. *J. Clin. Periodontol.* 25 134–144. 10.1111/j.1600-051X.1998.tb02419.x 9495612

[B115] Sokol H. Leducq V. Aschard H. Pham H. P. Jegou S. Landman C. (2017). Fungal microbiota dysbiosis in IBD. *Gut* 66 1039–1048. 10.1136/gutjnl-2015-310746 26843508 PMC5532459

[B116] Sousa E. L. Martinho F. C. Leite F. R. Nascimento G. G. Gomes B. P. (2014). Macrophage cell activation with acute apical abscess contents determined by interleukin-1 Beta and tumor necrosis factor alpha production. *J. Endod.* 40 1752–1757. 10.1016/j.joen.2014.06.019 25205261

[B117] Stecher B. Hardt W. D. (2011). Mechanisms controlling pathogen colonization of the gut. *Curr. Opin. Microbiol.* 14 82–91. 10.1016/j.mib.2010.10.003 21036098

[B118] Swidsinski A. Khilkin M. Kerjaschki D. Schreiber S. Ortner M. Weber J. (1998). Association between intraepithelial *Escherichia coli* and colorectal cancer. *Gastroenterology* 115 281–286. 10.1016/S0016-5085(98)70194-5 9679033

[B119] Taieb F. Nougayrede J. P. Watrin C. Samba-Louaka A. Oswald E. (2006). *Escherichia coli* cyclomodulin Cif induces G2 arrest of the host cell cycle without activation of the DNA-damage checkpoint-signalling pathway. *Cell Microbiol.* 8 1910–1921. 10.1111/j.1462-5822.2006.00757.x 16848790

[B120] Teles R. Teles F. Frias-Lopez J. Paster B. Haffajee A. (2013). Lessons learned and unlearned in periodontal microbiology. *Periodontol* 62 95–162. 10.1111/prd.12010 23574465 PMC3912758

[B121] Temoin S. Chakaki A. Askari A. El-Halaby A. Fitzgerald S. Marcus R. E. (2012). Identification of oral bacterial DNA in synovial fluid of patients with arthritis with native and failed prosthetic joints. *J. Clin. Rheumatol.* 18 117–121. 10.1097/RHU.0b013e3182500c95 22426587 PMC3888235

[B122] Tjalsma H. Boleij A. Marchesi J. R. Dutilh B. E. (2012). A bacterial driver-passenger model for colorectal cancer: beyond the usual suspects. *Nat. Rev. Microbiol.* 10 575–582. 10.1038/nrmicro2819 22728587

[B123] Tlaskalova-Hogenova H. Stepankova R. Hudcovic T. Tuckova L. Cukrowska B. Lodinova-Zadnikova R. (2004). Commensal bacteria (normal microflora), mucosal immunity and chronic inflammatory and autoimmune diseases. *Immunol. Lett.* 93 97–108. 10.1016/j.imlet.2004.02.005 15158604

[B124] Tlaskalova-Hogenova H. Vannucci L. Klimesova K. Stepankova R. Krizan J. Kverka M. (2014). Microbiome and colorectal carcinoma: insights from germ-free and conventional animal models. *Cancer J.* 20 217–224. 10.1097/PPO.0000000000000052 24855011

[B125] Tomas I. Diz P. Tobias A. Scully C. Donos N. (2012). Periodontal health status and bacteraemia from daily oral activities: systematic review/meta-analysis. *J. Clin. Periodontol.* 39 213–228. 10.1111/j.1600-051X.2011.01784.x 22092606

[B126] Toprak N. U. Yagci A. Gulluoglu B. M. Akin M. L. Demirkalem P. Celenk T. (2006). A possible role of *Bacteroides fragilis* enterotoxin in the aetiology of colorectal cancer. *Clin. Microbiol. Infect.* 12 782–786. 10.1111/j.1469-0691.2006.01494.x 16842574

[B127] Trojanowska D. Zwolinska-Wcislo M. Tokarczyk M. Kosowski K. Mach T. Budak A. (2010). The role of Candida in inflammatory bowel disease. Estimation of transmission of *C. albicans* fungi in gastrointestinal tract based on genetic affinity between strains. *Med. Sci. Monit.* 16 Cr451–Cr457. 20885347

[B128] Tsoi H. Chu E. S. H. Zhang X. Sheng J. Nakatsu G. Ng S. C. (2017). *Peptostreptococcus anaerobius* induces intracellular cholesterol biosynthesis in colon cells to induce proliferation and causes dysplasia in mice. *Gastroenterology* 152 1419–1433. 10.1053/j.gastro.2017.01.009 28126350

[B129] Underhill D. M. Iliev I. D. (2014). The mycobiota: interactions between commensal fungi and the host immune system. *Nat. Rev. Immunol.* 14 405–416. 10.1038/nri3684 24854590 PMC4332855

[B130] Ungprasert P. Wijarnpreecha K. Wetter D. A. (2017). Periodontitis and risk of psoriasis: a systematic review and meta-analysis. *J. Eur. Acad. Dermatol. Venereol.* 31 857–862. 10.1111/jdv.14051 27862342 PMC5408312

[B131] Urzúa B. Hermosilla G. Gamonal J. Morales-Bozo I. Canals M. Barahona S. (2008). Yeast diversity in the oral microbiota of subjects with periodontitis: *Candida albicans* and *Candida dubliniensis* colonize the periodontal pockets. *Med. Mycol.* 46 783–793. 10.1080/13693780802060899 18608938

[B132] Vannucci L. Stepankova R. Kozakova H. Fiserova A. Rossmann P. Tlaskalova-Hogenova H. (2008). Colorectal carcinogenesis in germ-free and conventionally reared rats: different intestinal environments affect the systemic immunity. *Int. J. Oncol.* 32 609–617. 10.3892/ijo.32.3.609 18292938

[B133] Wade W. G. (2013). The oral microbiome in health and disease. *Pharmacol. Res.* 69 137–143. 10.1016/j.phrs.2012.11.006 23201354

[B134] Walker A. W. Ince J. Duncan S. H. Webster L. M. Holtrop G. Ze X. (2011). Dominant and diet-responsive groups of bacteria within the human colonic microbiota. *ISME J.* 5 220–230. 10.1038/ismej.2010.118 20686513 PMC3105703

[B135] Wickstrom C. Davies J. R. Eriksen G. V. Veerman E. C. Carlstedt I. (1998). MUC5B is a major gel-forming, oligomeric mucin from human salivary gland, respiratory tract and endocervix: identification of glycoforms and C-terminal cleavage. *Biochem. J.* 334(Pt 3), 685–693. 10.1042/bj3340685 9729478 PMC1219739

[B136] Wong B. K. McGregor N. R. Butt H. L. Knight R. Liu L. Y. Darby I. B. (2016). Association of clinical parameters with periodontal bacterial haemolytic activity. *J. Clin. Periodontol.* 43 503–511. 10.1111/jcpe.12554 27105613

[B137] Wu G. D. Chen J. Hoffmann C. Bittinger K. Chen Y. Y. Keilbaugh S. A. (2011). Linking long-term dietary patterns with gut microbial enterotypes. *Science* 334 105–108. 10.1126/science.1208344 21885731 PMC3368382

[B138] Yamaoka Y. Suehiro Y. Hashimoto S. Hoshida T. Fujimoto M. Watanabe M. (2017). *Fusobacterium nucleatum* as a prognostic marker of colorectal cancer in a Japanese population. *J. Gastroenterol.* 53 517–524. 10.1007/s00535-017-1382-6 28823057

[B139] Yu T. C. Guo F. F. Yu Y. N. Sun T. T. Ma D. Han J. X. (2017). *Fusobacterium nucleatum* promotes chemoresistance to colorectal cancer by modulating autophagy. *Cell* 170 548–563. 10.1016/j.cell.2017.07.008e16 28753429 PMC5767127

[B140] Zanzoni A. Spinelli L. Braham S. Brun C. (2017). Perturbed human sub-networks by *Fusobacterium nucleatum* candidate virulence proteins. *Microbiome* 5:89. 10.1186/s40168-017-0307-1 28793925 PMC5551000

[B141] Zhou Y. J. Gao H. Y. Mihindukulasuriya K. A. La Rosa P. S. Wylie K. M. Vishnivetskaya T. (2013). Biogeography of the ecosystems of the healthy human body. *Genome Biol.* 14:R1. 10.1186/Gb-2013-14-1-R1 23316946 PMC4054670

